# Sealing of adhesive systems in ferric sulfate-contaminated dentinal margins in class V composite resin restorations

**DOI:** 10.15171/joddd.2016.003

**Published:** 2016-03-16

**Authors:** Niloofar Shadman, Shahram Farzin Ebrahimi, Najmeh Mollaie

**Affiliations:** ^1^Assistant Professor, Department of Operative Dentistry, Faculty of Dentistry, Kerman University of Medical Sciences, Kerman, Iran; ^2^Assistant Professor, Department of Orthodontics, Faculty of Dentistry, Rafsanjan University of Medical Sciences, Rafsanjan, Iran

**Keywords:** Adhesive, composite restoration, hemostatic agent, microleakage

## Abstract

***Background.*** Hemostatic agents are applied to prepare an isolated bleeding-free condition during dental treatments and can influence adhesive restorations. This study evaluated the effect of a hemostatic agent (ViscoStat) on microleakage of contaminated dentinal margin of class V composite resin restorations with three adhesives.

***Methods.*** Sixty freshly extracted human molars were selected and class V cavities (3×3×1.5 mm) were prepared on buccal and lingual surfaces. Gingival margins of the cavities were placed below the cementoenamel junction. The teeth were divided into six groups randomly. The adhesives were Excite, AdheSE and AdheSE One. In three groups, the gingival walls of the cavities were contaminated with ViscoStat and then rinsed. The cavities were restored with composite resin and light-cured. After storage in distilled water (37°C) for 24 hours and polishing, the samples were thermocycled and sealed with nail varnish. Then they were stored in 1% basic fuchsin for 24 hours, rinsed and mounted in self-cured acryl resin, followed by sectioning buccolingually. Dye penetration was observed under a stereomicroscope and scored. Data were statistically analyzed with Kruskal-Wallis and Mann-Whitney U tests. P<0.05 was set as the level of significance.

***Results.*** Only in the Excite group, contamination did not have adverse effects on dentin microleakage (P > 0.05). In the contaminated groups, Excite had significantly less microleakage than the others (P = 0.003). AdheSE and AdheSE One did not exhibit significant difference in microleakage (P > 0.05).

***Conclusion.*** ViscoStat hemostatic agent increased dentinal microleakage in AdheSE and AdheSE One adhesives with no effect on Excite.

## Introduction

 There has been an increasing tendency to use composite resins in dentistry in recent years, which might be attributed to their benefits, especially esthetics, minimum tooth preparation and preservation of tooth structure.^1^ They are suggested for class V restorations, where esthetic is a critical concern.

 Microleakage is a clinically undetectable passage of molecules, ions, bacteria, bacterial products and fluids through tooth-restoration interfaces and is a main criterion for evaluating the success rate of restorative materials. Up to now, no restorative material capable of preventing leakage at cervical margins has been presented. Secondary caries, as a consequence of microleakage, is the most common cause of composite resin restoration replacements.^2^

 Isolation from blood, saliva, etc is very critical because they can have adverse effects on hybrid layer formation and adhesives’ sealing and they can induces gap formation and microleakage.^3^

 Use of hemostatic agents is indicated in class V cavities near the gingival margins, cementation of all-ceramic crowns, subgingival tooth preparations and interproximal areas with chronic irritation and inflammation with gingival bleeding.^3^ These agents have various formulations and effects, for example aluminum chloride (affects collagen fibers around damaged capillaries and contract them),^4^ ferric sulfate and iron solutions (can distort blood proteins) and 0.1% epinephrine (contracts the muscles of vessels). They can produce a properly isolated condition.^3,5^

 Because of hydrophilic characteristics of hemostatic materials, they can contaminate every stage of adhesives and thereby have adverse effects on bonding quality.^3^Hemostatic agents should be used in cases with gingival bleeding or subgingival margins.

 Some studies have revealed that contamination with hemostatic agents can decrease composite resin bond strength;^6,7^ however, this issue is controversial.^8^ Another study showed that contamination with hemostatic agents did not affect the microleakage of a two-step self-etch adhesive in class V cavities.^9^

 Therefore in cases with gingival bleeding or subgingival margins, the efficacy of self-etch and etch-and-rinse adhesive systems should be considered and tested first and then they can be recommended for clinical practice.

 The aim of the present study was to evaluate the influence of ferric sulfate (a hemostatic agent) on the microleakage of dentinal margins in class V composite resin restorations. The null hypothesis was that ViscoStat hemostatic agent did not have any effect on microleakage of etch-and-rinse and self-etch adhesives in class V composite resin restorations.

## Methods

 This study was approved in Kerman Research Center Committee of Ethics. In this in vitro study, sixty freshly extracted caries-free human third molars were cleaned up of calculus, debris and soft tissues, stored for 24 hours in a solution of 0.5% chloramin-T (Fisher Chemical, Fair Lawn, NJ, USA) and rinsed with saline solution. A total of 120 Class V cavities were prepared on the buccal and lingual surfaces of the samples by using a diamond bur (SS White, Great White Series, Lakewood, NJ, USA) with air-water coolant spray. Cavity dimensions were standardized (3×3×1.5 mm) with a periodontal probe. The occlusal enamel margins were beveled with a 1-mm width at a 45° angle and the gingival margins were placed beyond the CEJ (1 mm). One operator prepared all the cavities. The teeth were divided into six groups randomly (n = 20). The materials were used according to the manufacturers’ instructions (Table 1).

**Table 1:** Compositions of the materials used in this study

**Materials**	**Type**	**Composition**	**Manufacturer**	**Batch #**
**Excite**	Etch-and-rinse	phosphoric acid acrylate, HEMA, dimethacrylates, ethanol, silicon dioxide, photoinitiator	Ivoclar/Vivadent, Schaan, Liechtenstein	L19442
**AdheSE**	Two-Step Self-etch	Adhesive: dimethacrylate, phosphoric acid acrylate, initiators and stabilizers. Bonding: HEMA, dimethacrylate, silicon dioxide, initiators and stabilizers.	Ivoclar/Vivadent, Schaan, Liechtenstein	adhesive: M02841; bonding: L49735
**AdheSE- One**	One-step Self-etch	Derivatives of bis-acrylamide, water, bis-methacrylamidedihydrogen phosphate, amino acid acrylamide, hydroxy alkyl methacrylamid, highly dispersed silicon dioxide, catalysts and stabilizers.	Ivoclar/Vivadent, Schaan, Liechtenstein	L42998
**ViscoStat**	Hemostatic agent	A viscous 20% ferric sulfate coagulative hemostatic gel.	Ultradent Product Inc., Utah, USA	B2P7H
**Inten-S**	Light-Cure Composite	Filler Composition: barium glass, silica, titanium oxide Matrix composition: Bis-GMA, UDMA, Bis EMA6	Ivoclar/Vivadent, Schaan, Liechtenstein	J21793
**N-Etch**	Etchant	Phosphoric acid (37 wt% in water) ,thickeners and pigments	Ivoclar/Vivadent, Schaan, Liechtenstein	L37473

**Group 1:** ViscoStat (Ultradent Product Inc., Utah, USA) is a ferric sulfate compound that was placed in the gingival walls for two minutes and then rinsed thoroughly for 60 seconds. The cavities were etched with 37% phosphoric acid (Ivoclar/Vivadent, Schaan, Liechtenstein) for a minimum of 15 seconds in enamel and a maximum of 15 seconds in dentin and rinsed for 30 seconds under running water. The cavities were blot-dried, followed by application of one coat of Excite (Ivoclar/Vivadent, Schaan, Liechtenstein), air-dried for 5 seconds and polymerized for 20 seconds with a quartz-tungsten-halogen (QTH) light-curing unit (Optilux 501, Demetron Kerr, Danbury, CT, USA) at a light intensity of 650 mW/cm^2^,which was monitored with a radiometer. Then the cavities were restored with microhybrid composite resin (Inten-S, Shade A1, Ivoclar) in three increments and each layer was light-cured for 40 seconds.

**Group 2:** No ViscoStat treatment was applied to the cavities prior to etching. Etching, bonding and restoring were carried out similar to those in group 1.

**Group 3:** After ViscoStat application similar to that in group 1, and rinsing and blot drying, the primer of AdheSE (Ivoclar/Vivadent, Schaan, Liechtenstein) was applied for 30 seconds to the cavity and after air drying, the adhesive of AdheSE was applied for 20 seconds, air-dried, light-cured 20 seconds and restoration was carried out similar to that in group 1.

**Group 4:** No ViscoStat was applied to the cavities. AdheSE bonding system was used similar to that in group 3 and restorative procedures were carried out similar to those in group 1.

**Group 5:** After ViscoStat application, similar to that in group 1, rinsing and blot drying, one coat of AdheSE One (Ivoclar/Vivadent, Schaan, Liechtenstein) adhesive was applied for 30 seconds to the cavity, gently air-dried, light-cured for 20 seconds and restoration was carried out similar to that in group 1.

**Group 6:** No ViscoStat was applied to the cavities. AdheSE One bonding system was used similar to that in group 5 and restored similar to that in group 1.

 Then the teeth were restored for 24 hours in 37°C distilled water. Restorations were finished with knife-edge finishing burs and polished with Sof-Lex (3M, ESPE, St Paul, MN, USA) aluminum oxide disks. Thermal cycling was carried out for 500 cycles at 5/55°C (a dwell time of 60 seconds and a transfer time 15 seconds). The root apices were sealed with sticky wax and two layers of nail varnish were applied to the entire teeth surface of teeth except for the restorations and 1 mm surrounding them. The samples were immersed in a 1% basic fuchsin solution for 24 hours and then washed under running water.

 After mounting the specimens in auto-cured clear acrylic resin (Castin Craft), they were sectioned with a low-speed diamond disk (FEJ, Germany) buccolingually through the center of class V restorations. Two blinded examiners evaluated sections at ×40 under a stereomicroscope (Carl Zeiss, SAS, Germany) for dye penetration. Microleakage was scored on a 0‒3 scale:

 0: no dye penetration

 1: dye penetration up to less than half the axial wall

 2: dye penetration up to more than half the axial wall

 3: dye penetration to the axial wall and along the axial wall

 The association between the treatment with ViscoStat and microleakage values was analyzed by Kruskal-Wallis test; then the groups were analyzed with Mann-Whitney U test at P < 0.05 as the level of significance to evaluate differences between the groups.

## Results

 None of the adhesive systems used in this study completely prevented microleakage at enamel and dentin margins (Table 2). The mean ranks of microleakage values in dentinal margins in ViscoStat-applied groups are presented in Table 3. Kruskal-Wallis test showed significant differences in dentinal margin microleakage (P = 0.003) and Excite had the lowest microleakage score than the others. Comparisons between the groups are presented in Table 4.

**Table 2:** Frequency of microleakage scores at dentin and enamel margins

**Groups**	**Scores**							
	**Dentin**				**Enamel**			
	**Score 0**	**Score 1**	**Score 2**	**Score 3**	**Score 0**	**Score 1**	**Score 2**	**Score 3**
**VS** ^*^ **+EX** ^†^	6	1	5	8	-	-	-	-
**EX**	3	7	4	6	15	5	0	0
**VS+Ad** ^‡^	0	2	1	17	-	-	-	-
**Ad**	11	1	3	5	10	6	4	0
**VS+Ad-One** ^#^	0	1	4	15	-	-	-	-
**Ad-One**	0	13	2	5	2	15	3	0

^*^VS: ViscoStat, ^†^EX: Excite, ^‡^Ad: AdheSE, ^#^Ad-One: AdheSE One

**Table 3:** Mean rank values of microleakage in dentinal margins of ViscoStat applied groups

**Group**	**Mean Rank**	**Chi-squared**	**df**	**P-value**
**Dentin-1**	21.55			
**Dentin-3**	36.05	11.50	2	0.003
**Dentin-5**	33.90			

**Table 4:** Mann-Whitney U test for comparison among ViscoStat applied groups

**Group**	**Groups 1-3**	**Groups 1-5**	**Groups 3-5**
**Dentin**	P = 0.003	P = 0.01	P = 0.52
	P < 0.05	P < 0.05	P > 0.05

 Comparisons between non-ViscoStat-applied groups (2, 4 and 6) with Kruskal-Wallis test showed no significant differences at dentinal margin microleakage (P > 0.05).

 There were no significant differences in microleakage values at the gingival margins of the Excite groups (P = 0.75), but at gingival margins of AdheSE (P < 0.001) and AdheSE One (P < 0.001) groups there were significant differences, with more microleakage in the ViscoStat-applied groups than the non-ViscoStat applied groups.

 In relation to the dentinal margins of the non-ViscoStat-applied groups, there were no significant differences between them (P = 0.14) but AdheSE exhibited the least microleakage value. In comparison of enamel and dentinal margins of the non-ViscoStat-applied groups, in all the groups microleakage at enamel margins was less than that at dentinal margins; however, there was statistically significant difference only in Excite (P = 0.001). In relation to microleakage at enamel margins, there were no significant differences between the three groups (P = 0.23). Excite exhibited the lowest and AdheSE One exhibited the highest microleakage scores.

## Discussion

 Generally, all the hemostatic agents can contaminate each bonding step and remain in the adhesive or adhesive layers or between composite resin increments, promoting inadequate adaptation.^10^ViscoStat is a compound of ferric sulfate that causes blood clotting in a few seconds and acts as a clotting agent.^11^

 The null hypothesis was confirmed only for Excite, i.e. contamination with 20% ferric sulfate had no effect on cervical microleakage.

 Phosphoric acid (because of its strong acidity, pH=0.5) can remove surface ViscoStat contamination and many surface contaminants.^12^On the other hand, rinsing the cavity alone and also weak acidity of self-etch adhesives could not remove surface contamination, and residual ViscoStat prevented proper penetration of AdheSE and AdheSE One, with an adverse effect on marginal seal.

 In our previous study which was carried out on the effect of ViscoStat contamination on bond strength, it was concluded that contamination did not reduce shear bond strength of etch-and-rinse and one-step self-etch adhesives to dentin.^12^

 In SEM-EDX study of Ayo-Yusuf et al,^13^ which was carried out on dentin surface contaminated with some hemostatic agents, it appeared hemostatic agents removed the smear layer, which obstructs the orifices of dentinal tubules; in addition, because of their acidic pH (0.8-3) they formed an amorphous layer or a granular precipitate on the surface. Some precipitates are more soluble and rinsable and 37% phosphoric acid, in some kinds of hemostatic agents, can eliminate this amorphous layer but in others, H^+^ ions cannot penetrate into the depth of dentin and the dissolution of amorphous layer is limited.

 According to Land et al,^14^ 15.5% ferric sulfate solution can occlude dentinal tubule orifices and disturb etching and bonding processes.

 Kimmes et al^15^ demonstrated that after removing dentin contamination with ViscoStat and ViscoStat Plus (Ultradent Product Inc., Utah, USA) with water spray, there was no significant decrease in shear bond strength of Optibond Solo Plus (Kerr Corporation, Orange CA, USA). Hemostatic agent's acidity can produce some changes in dentin structure, and according to manufacturer's instructions, an 8-minute contact is necessary between ViscoStat and dentin for removing the smear layer, although the presence of other ingredients such as coating agent and the bonding agent can reduce the effect of H^+^ ions on the smear layer.

 In this study, comparison of the results of dentin microleakage in non-contaminated groups showed no significant differences among groups; some studies have concluded that bonding quality in etch-and-rinse and self-etch adhesives is very similar and self-etch adhesives have reduced clinical procedures.^16,17^

 Some investigators have reported that the presence of water in adhesive ingredients (such as AdheSE and AdheSE One) is an advantage because water rehydrates dentin and helps proper penetration of adhesives into collapsed collagen network.^18^On the other hand, Excite is very sensitive to the amount of substrate wetness and is technique-sensitive because it contains ethanol as a solvent.^19^

 Gagliardi et al^20^ demonstrated that Excite has relatively high values of dentin microleakage equal to self-etch adhesives, consistent with our results.

 In our study there were significant differences between the enamel and dentin margin microleakage values in the Excite group (P < 0.05). The greater microleakage value in dentin than enamel is related to differences in their compositions and structures. Adhesion to enamel is a relatively simple process without technical difficulties because enamel has a hypermineralized structure (90% vol: hydroxyapatite), while adhesion to dentin is more difficult because of higher amount of water and organic materials that can disrupt bonding process.^21^

 In other studies which compared self-etch and etch-and-rinse adhesives, microleakage in enamel was less than that at dentin margins.^22,23^

 One disadvantage of self-etch adhesives is that they may be ineffective in etching thick smear layer or prismless enamel. Resin tags obtained from etching enamel with phosphoric acid are thicker and uniform, but resin tags obtained after using self-etch adhesives are thin and are not uniform. Therefore, enamel microleakage in self-etch adhesives is higher.^24^ These findings are consistent with our study.

 Self-etch adhesives have shallower etching pattern because of weaker penetration of acidic adhesives to enamel porosities. Since acidic adhesives are not washed during the bonding procedure, they release calcium and phosphate ions from hydroxyapatite crystals into the adhesive. Elevated concentrations of phosphate and calcium ions can limit further dissolution of apatite, hence reducing enamel demineralization.^25^

 According to Owens et al,^26^ microleakage in etch-and-rinse adhesives is less than that in a lot of self-etch adhesives at enamel margins.

 Problems related to etching efficacy in self-etch adhesives are more common in ones with mild to moderate pH; nevertheless, with production of more acidic formulations, adhesion to enamel is more acceptable.^27^

 According to this study, ViscoStat hemostatic agent did not have any adverse effect on Excite dentinal microleakage but increased dentinal microleakage in AdheSE and AdheSE One groups. Use of etch-and-rinse adhesive systems is advocated with application of ViscoStat hemostatic agent.

## Acknowledgments

 The authors thank Kerman University of Medical Sciences for financial support and Miss Sadeghi for statistical analysis and Mrs Memaran Dadgar.

## Authors’ contributions

 NS &amp; SFE contributed to the concept and the design of the study, as well as definition of intellectual content. All authors contributed to the literature review, and collectively performed the experimental studies. NS &amp; NM were responsible for the acquisition of the data. NS was responsible for the statistical analysis with the supervision of the statistician. All authors contributed to the drafting and critical revision of the manuscript, and have read and approved the final manuscript.

## Funding

 Kerman University of Medical Sciences was the source of financial support and funding.

## Competing interests

 The authors declare that they have no competing interests with regards to authorship and/or publications of this paper.

## Ethics approval

 This study was approved in Kerman University of Medical Sciences Research Center Committee of Ethics.
